# The complete chloroplast genome of *Uvaria macrophylla* Roxb. (Annonaceae)

**DOI:** 10.1080/23802359.2019.1659108

**Published:** 2019-09-17

**Authors:** Qiaohua Wu, Xiaojuan Liu, Yile Chen, Deyu Zhang, Seping Dai

**Affiliations:** a Guangzhou Institute of Forestry and Landscape Architecture, Guangzhou, China;; b Landscaping company of Guangzhou, Guangzhou, China;; c The Affiliated Taihe Experimental School of South China Normal University, Guangzhou, China;; d Xia Mao Elementary School, Guangzhou, China

**Keywords:** Uvaria macrophylla, complete chloroplast genome, automated assembly, phylogenetic analysis

## Abstract

*Uvaria macrophylla* (Annonaceae) is an erect shrub with multiple medicinal properties. In this study, we report the complete chloroplast genome of *U. macrophylla*, assembled from whole-genome high-throughput sequencing reads, as a resource for future studies on the phylogeny and evolution of Annonaceae. The chloroplast genome was 192,782 bp in length, with a large single-copy (LSC) region of 83,581 bp, a small single-copy (SSC) region of 3,741 bp, separated by two inverted repeat (IR) regions of 52,730 bp each. It was predicted to contain 151 genes, with an overall GC content of 38.7%. Phylogenetic analysis of 105 protein-coding sequences of 13 plant plastomes showed that *U. macrophylla* is closest to *Annona cherimola*.

*Uvaria macrophylla*, an erect shrub, belongs to the family Annonaceae. *U. macrophylla* is widely distributed in Hainan, Guangdong and Guangxi provinces of southern China, occurring in low altitude boscage and woodland (Wu et al. 2004). The medicinal properties of *U. macrophylla* has been widely studied by scientists (Zafra Polo et al. 1998; Wang et al. 2005; Zhu et al. 2011; Chen et al. 2012). Some studies revealed that the constituents in the extracts from *U. macrophylla* exhibited inhibitory activities toward a number of human cancer cell lines (Wang et al. 2002, 2003; Zhang et al. 2002). In this study, we characterized the complete chloroplast genome sequence of *U. macrophylla* as a resource for future genetic studies on this plant and its related species.

Leaf samples of *U. macrophylla* were obtained from the Guangzhou Institute of Forestry and Landscape Architecture, with voucher specimen (SYS-Bore-2019-04) being deposited at Sun Yat-sen University Herbarium (SYS). After DNA extraction, a ∼350-bp insertion library was constructed and then sequenced (paired-end 150 bp) on the Illumina Hiseq Xten platform, generating approximately 4 Gb raw data. Using a partial *rbcL* gene from *U. macrophylla* (KP094324) as a seed, all data were assembled into an intact circular chloroplast genome using the software NOVO Plasty (Dierckxsens et al. 2017). The assembled chloroplast genome sequence was annotated using DOGMA (Wyman et al. 2004) and manually corrected.

The complete chloroplast genome sequence of *U. macrophylla* (GenBank accession MH992130) was 192,782 bp in length, with a large single-copy (LSC) region of 83,581 bp, a small single-copy (SSC) region of 3,741 bp, and two inverted repeat (IR) regions of 52,730 bp each. A total of 151 genes were predicted, consisting of 105 protein-coding genes, 38 tRNA genes, and 8 rRNA genes. The overall GC content was 38.7%.

To understand the phylogenetic position of *U. macrophylla*, an ML tree was drawn for *U. macrophylla* and 12 other representative species from Magnoliales, Ranales, Sapindales and Aristolochiales. The homologous blocks in the chloroplast genomes among these 13 species were extracted using HomBlocks tool (Bi et al. 2018). These blocks were then aligned using MAFFT v7.307 (Katoh and Standley 2013), and RaxML (Stamatakis, 2014) was used to construct a maximum likelihood tree with *C. caroliniana* as outgroup. As shown in Figure 1, *U. macrophylla* is closest to *Annona cherimola* from the same family.

**Figure 1.**
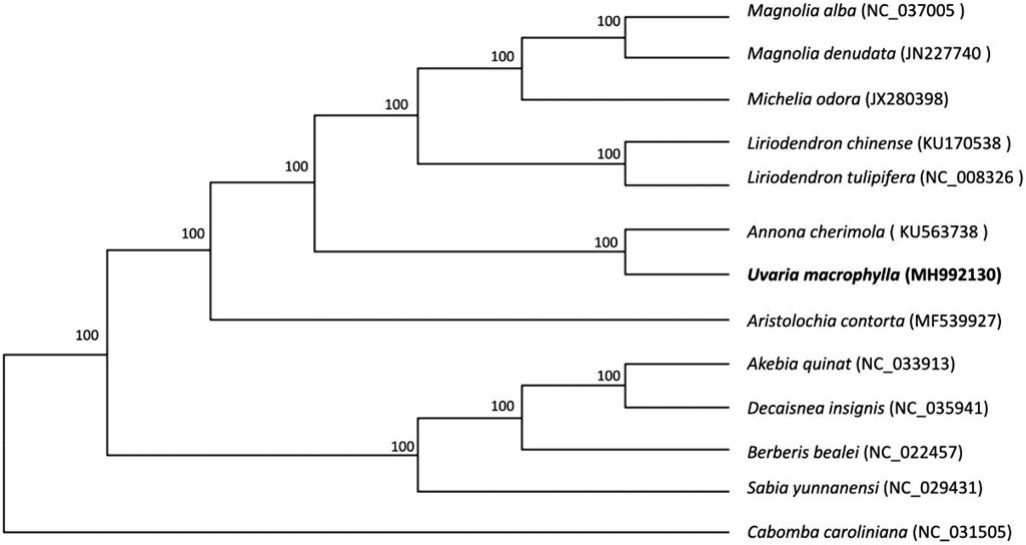
Maximum likelihood tree of *U. macrophylla* and 12 other related species based on homologous blocks of whole chloroplast genome sequence, with *C. caroliniana* as out group. Bootstrap support values (based on 1000 replicates) are shown next to the nodes.

## References

[CIT0001] Bi G, Mao Y, Xing Q, Cao M 2018 A multiple-alignment construction pipeline for organelle phylogenomics based on locally collinear block searching. Genomics. 110:18–22. 28780378 10.1016/j.ygeno.2017.08.001

[CIT0002] Chen Z, Liu XR, Liu YL, et al. 2012 ChemInform Abstract: Two New Flavones from *Uvaria macrophylla* Roxb. var. microcarpa and Their Cytotoxic Activities. Cheminform. 43:2857–2863.

[CIT0003] Dierckxsens N, Mardulyn P, Smits G 2017 NOVOPlasty: de novo assembly of organelle genomes from whole genome data. Nucleic Acids Res. 45:e18. 28204566 10.1093/nar/gkw955 PMC5389512

[CIT0004] Katoh K, Standley DM 2013 MAFFT multiple sequence alignment software version 7: Improvements in performance and usability. Mol Biol Evol. 30:772–780. 23329690 10.1093/molbev/mst010 PMC3603318

[CIT0005] Stamatakis A 2014 RAxML version 8: a tool for phylogenetic analysis and post analysis of large phylogenies. Bioinformatics. 30:1312–1313. 24451623 10.1093/bioinformatics/btu033 PMC3998144

[CIT0006] Wang S, Zhang PC, Chen RY, et al. 2002 A Novel Dihydro flavone from the Roots of *Uvaria Macrophylla*[J]. Chinese Chem Lett. 13:857–858.

[CIT0007] Wang S, Chen RY, Yu SS, Yu DQ 2003 Uvamalols D-G: novel polyoxygenated seco-cyclohexenes from the roots of Uvaria macrophylla. J Asian Nat Prod Res. 5:17–23. 12608634 10.1080/1028602031000080414

[CIT0008] Wang S, Zhang PC, Chen RY, Dai SJ, Yu SS, Yu DQ 2005 Four new compounds from the roots of *Uvaria macrophylla*. J Asian Nat Prod Res. 7:687–694. 16176900 10.1080/10286020412331286524

[CIT0009] Wu ZY, Raven P, Hong DY, et al. 2004 Flora Reipublicae Popularis Sinicae. Beijing (China): Science Press (in Chinese), p. 126.

[CIT0010] Wyman SK, Jansen RK, Boore JL 2004 Automatic annotation of organellar genomes with DOGMA. Bioinformatics. 20:3252–3255. 15180927 10.1093/bioinformatics/bth352

[CIT0011] Zafra Polo MC, Figadère B, Gallardo T, et al. 1998 Natural acetogenins from Annonaceae, synthesis and mechanisms of action. Phytochemistry. 48:1087–1117.

[CIT0012] Zhang HL, Wang S, Chen RY, Yu DQ 2002 Studies on chemical constituents of Uvaria macrophylia. Yao Xue Xue Bao. 37:124–127. 12579957

[CIT0013] Zhu XY, Lin SW, Lu RM 2011 Analysis of chemical components of volatile oils from leaves of *Uvaria microcarpa* Champ.ex Benth by SFE-CO_2_. Medicinal Plant. 39:13376–13377.

